# Comparison Between Shoulder Flexed and Extended Positions in Elbow Flexion Resistance Training on Regional Hypertrophy and Maximum Strength: Preacher versus Bayesian Cable Curls

**DOI:** 10.1002/ejsc.12279

**Published:** 2025-03-13

**Authors:** Parsa Attarieh, João Pedro Nunes, Saeed Khani, Saman Negahdar, Amirali Goli, Hamed Nazarirad, Shahriar Nazarirad, Shima Mojtahedi, Kazunori Nosaka, Rahman Soori

**Affiliations:** ^1^ Department of Exercise Physiology Faculty of Sport Sciences and Health University of Tehran Tehran Iran; ^2^ Physical Education and Sport Center Londrina State University Londrina Brazil; ^3^ School of Medical and Health Sciences Edith Cowan University Joondalup Australia; ^4^ Department of Biological Sciences in Sport Faculty of Sport Sciences and Health Shahid Beheshti University Tehran Iran; ^5^ Department of Physiology Division of Sports Physiology Faculty of Medicine Çukurova University Adana Turkey

**Keywords:** exercise selection, inhomogeneous hypertrophy, muscle architecture, strength training

## Abstract

In the present study, the effects of resistance training on regional hypertrophy and maximum strength of the elbow flexor muscles were compared between elbow flexion exercises performed with different shoulder joint angles (∼50° of flexion vs. extension) while matched for resistance profiles. In a within‐subject design, 15 young men (25.6 ± 2.1 y; 77.3 ± 6.8 kg; 175.1 ± 5.7 cm) underwent a resistance training program twice a week for 10 weeks (3–5 sets, 8–12RM), and their arms were dominant‐side balanced, randomly assigned to one of the two conditions according to elbow flexion exercises: unilateral cable curl with shoulder flexed (Preacher curl; PREA) or unilateral cable curl with shoulder extended (Bayesian curl; BAYE). B‐mode ultrasound imaging was used to measure changes in muscle thickness of the biceps brachii and brachialis at proximal, mid, and distal arm regions, and one‐repetition maximum tests were completed in each respective trained exercise before and after training. Both conditions showed significant increases in muscle thickness (*p* < 0.05) with no significant differences between them (*p* > 0.05) across the biceps brachii proximal, mid, and distal regions (relative change [Hedges' g effect size]; PREA: 6%[0.51], 7%[0.49], 7%[0.53]; BAYE: 9%[0.73], 9%[0.62], 9%[0.62]) and brachialis (PREA: 10%[0.72]; BAYE: 8%[0.65]). Similarly, significant improvements in maximum strength were observed (*p* < 0.05), with equivalent results between conditions (PREA: 28%[0.85], BAYE: 37%[1.22]; equivalence testing, *p*‐values = 0.061, 0.637). In conclusion, the shoulder joint angle does not seem to affect muscle hypertrophy and maximum strength gains after different elbow flexion exercises matched for resistance profiles.

**Summary**

- Training biceps curl with the shoulder flexed (PREA) or extended (BAYE) resulted in similar moderate‐to‐large increases in elbow flexor muscle thickness and maximum strength.
- Similar increases in muscle thickness were observed for all analyzed sites in both conditions, that is, no regional hypertrophy was observed.
- The biceps brachii do not seem to benefit from longer‐length resistance training nor exhibit inhomogeneous regional hypertrophy.

## 1 Introduction

Neuromuscular adaptations to resistance training (RT) programs are influenced by the manipulation of variables such as training intensity, volume, and frequency (B. Schoenfeld et al. 2021), as well as exercise selection, which is often overlooked (Kassiano et al. 2022). Exercises can be characterized according to several features (e.g., number of joints involved, apparatus used, and resistance profile), and recent findings indicate that improvements in muscle size and strength are affected by the exercised muscle length (Kassiano et al. 2023; Oranchuk et al. 2019).

Resistance exercises that stimulate the muscles at longer muscle lengths seem to evoke greater hypertrophy (Kassiano et al. 2023; Oranchuk et al. 2019; Ottinger et al. 2023). This can be easily accomplished by training in lengthened muscle positions either with partial range of motion exercises or isometric contractions (Kassiano et al. 2023; Oranchuk et al. 2019). However, the traditional dynamic full range of motion exercises is the basis of the RT prescription (B. Schoenfeld et al. 2021). In this sense, among the variety of exercises that can be done while training in the traditional form, opting for exercises that focus on longer muscle lengths is a practicable option. For bi‐articular muscles, this involves selecting exercises where the proximal joint of the target muscle remains in an anatomical or neutral position rather than a muscle‐shortened position—for example, plantar flexion with extended knee versus flexed knee (Kinoshita et al. 2023), or knee extension with extended hip versus flexed hip (Larsen et al. 2024)—or training in pre‐stretched positions rather than neutral (shorter) positions (Maeo et al. 2021, 2023; Stasinaki et al. 2018).

Although the literature on this topic is still limited, the benefits of longer‐length training appear to depend on the muscle analyzed. While the triceps surae, hamstrings, and quadriceps seem to experience greater growth with training at longer lengths, evidence regarding the biceps brachii remains conflicting (Kassiano et al. 2023; Ottinger et al. 2023). Shoulder extension lengthens the biceps brachii (Iwane et al. 2023), and training an elbow‐flexion exercise with the shoulder extended probably favors the hypertrophic response in comparison to training in a pre‐shortened position. Alternative hypotheses suggest that the biceps may not experience greater hypertrophy when training at longer muscle lengths because it cannot reach the very descending portion of the force–length curve (Ottinger et al. 2023). Another possibility is that after pre‐lengthening or pre‐shortening the biceps brachii (Murray et al. 2000), the brachialis may take on a greater role in the exercise, receiving a larger stimulus (Kawakami et al. 1994; Nosaka and Sakamoto 2001), and this shift could reduce potential differences in biceps growth between exercises. However, these hypotheses remain to be tested.

Previous studies in which researchers sought to explore the influence of training the biceps brachii in different lengths have limited generalizability. In studies where the effect of varying shoulder positions was analyzed, the compared exercises also differed in their resistance profiles (Costa et al. 2021; Kassiano et al. 2025; Korta et al. 2023; Vendruscolo et al. 2024); consequently, muscle length was not isolated as a factor, making it difficult to determine its specific impact on hypertrophy and strength adaptations. Moreover, in many cases, other compound exercises involving elbow flexion were included in the training program (Costa et al. 2021; Nunes et al. 2020; Vendruscolo et al. 2024), further introducing confounding factors. Finally, hypertrophy was often assessed considering changes in overall elbow flexor muscle thickness (measured as the distance between the most superficial muscular arm region and the bone) without distinguishing between the biceps brachii and brachialis (Costa et al. 2021; Kassiano et al. 2025; Korta et al. 2023; Sato et al. 2021; Vendruscolo et al. 2024). In such situations, it is not possible to determine whether the training effects on hypertrophy stem from the biceps or the brachialis. Isolating the biceps in measurements is essential for determining the effects of training it at different lengths, as well as exploring whether it exhibits inhomogeneous regional hypertrophy, as observed in other muscles. For instance, performing leg extensions with greater hip flexion has been shown to impair both acute and long‐term proxies of proximal rectus femoris hypertrophy (Mitsuya et al. 2023; Larsen et al. 2024). It remains to be investigated whether altering joint position affects the regional hypertrophy of other muscles.

**FIGURE 1:**
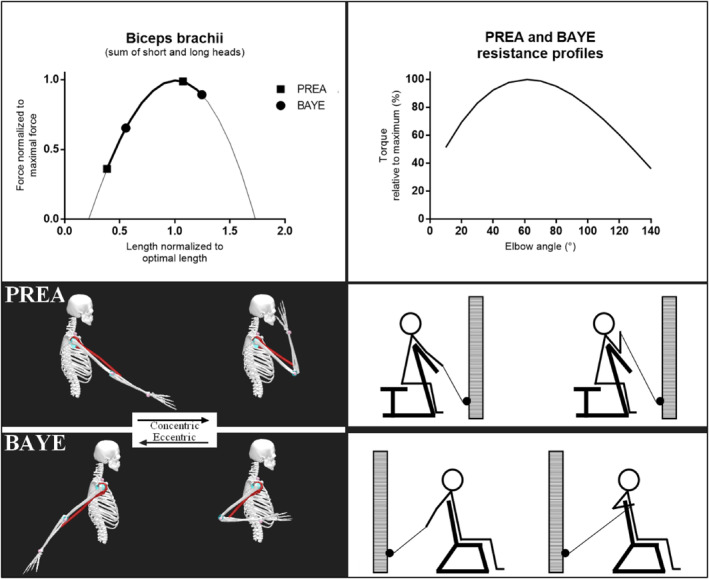
Top left: Biceps brachii normalized force–length curve and operating length for Preacher (PREA; shoulder flexed 50°) and Bayesian (BAYE; shoulder extended 50°) biceps curls during the 10°–140° range of motion. These were obtained using OpenSim Arm26 model (Holzbaur et al. 2005), considering biceps brachii as a single unit (sum of short and long heads) while maintaining relative contributions of each head to the length and active force and using muscle–tendon default parameters. Top right: Resistance profiles (relative torque to elbow angle) of the trained exercises. Bottom left and right: Example of how PREA (top) and BAYE (bottom) exercises were conducted during testing and training sessions. Note the same hand‐pulley distance at the start of the exercises and the same arm‐cable angles between exercises at the start and end of the range of motion. Estimated peak torques (arm‐cable angle: 90°) were at 61.5° elbow flexion.

Therefore, the objective of the present study was to compare the effects of elbow‐flexion exercise training on regional hypertrophy of biceps brachii and brachialis, and elbow flexion maximum strength gains after two exercise conditions matched for resistance profiles: unilateral Preacher cable curl (PREA), performed with the arms supported on an angled bench, keeping the shoulder in a flexed position; and unilateral Bayesian cable curl (BAYE), similar to the commonly known incline curl, performed with the shoulder in an extended position. Figure 1 illustrates how exercises were performed. It was hypothesized that BAYE would present advantages over PREA at increasing biceps brachii size (Kinoshita et al. 2023; Larsen et al. 2024; Maeo et al. 2021, 2023), whereas changes in strength would be equivalent between conditions (Maeo et al. 2021, 2023).

## 2 Methods

### 2.1 Experimental Design

A within‐subject design was carried out to compare the effects of training the biceps brachii in different shoulder positions on regional hypertrophy and strength gains. This design was chosen to eliminate any potential inter‐individual variations on training effects such as dietary intake, responsiveness, and training tolerance. This study is part of a larger research project designed to analyze the effects of RT in young male adults, but the only elbow flexion exercise was the biceps curl. The subjects' dominant arms (preferred hand for writing) were randomly assigned to one of the two conditions based on elbow flexor exercises, whereas the nondominant arms were assigned to the other: PREA or BAYE. Therefore, a balanced distribution of dominant and nondominant arms was achieved across the exercise conditions.

The current investigation was executed as follows: 1 week was used for pre‐training outcome assessments, 10 weeks for the progressive training program, and one more week for post‐training testing (at least 72 h after the last training session). Muscle hypertrophy was defined as changes in muscle thickness (MT) via ultrasonography, and muscular strength was assessed via one‐repetition maximum (1RM) tests in the respective trained exercises. Testing and training sessions were supervised by at least two researchers with international personal trainer certifications (1:2 participant:professional ratio) to standardize exercise techniques and help ensure the safety of the subjects. A 40‐g dose of concentrated protein powder (Milk Protein Pro Milk, Kalleh, Iran) was provided to the subjects after every training session as a strategy to increase their average daily protein intake and maximize the training responses (Morton et al. 2018). This investigation was conducted according to the Declaration of Helsinki and was approved by the University Ethics Committee.

### 2.2 Subjects

Recruitment was carried out through social media and home delivery of flyers in the university area. Interested subjects completed detailed health history and physical activity questionnaires, and were subsequently admitted if they met the following inclusion criteria: men, 18–35 years old, free from cardiac, orthopedic, or musculoskeletal disorders that could impede exercise practice, not consume drug or supplement ergogenic aids, and not be involved in the practice of RT over the 6 months before the start of the study. Of the 38 volunteers, 21 met the criteria, were evaluated at baseline, and began the training program. An attendance rate of less than 80% was set as an exclusion criterion, resulting in the exclusion of six participants. Fifteen individuals completed the study and were included in final analyses (age = 25.6 ± 2.1 years; body mass = 77.3 ± 6.8 kg; stature = 175.1 ± 5.7 cm; BMI = 25.2 ± 3.8 kg/m^2^), attending the necessary sample size defined a priori (*n* ≥ 15 per group) to achieve a power of 0.8 and an *α* of 0.05 for a moderate effect size of 0.55 in a two‐group, two‐time point design, for improving muscle size (B. J. Schoenfeld et al. 2017). Subjects were instructed to avoid changes in their habitual recreational physical activities and dietary intake and not to start taking any other nutritional supplements that could alter their performance during the study period. Written informed consent was obtained from all subjects after a detailed description of study procedures was provided.

### 2.3 MT Assessment

Measures of biceps brachii and brachialis MT were obtained using a B‐mode ultrasound machine with an 8–12 MHz linear probe (L741; SonoScape E1 EXP, China) by the same experimenter, blinded to condition allocation. Subjects were instructed to show up to the laboratory in the morning hours fasting for at least 8 h and not perform vigorous exercise for the previous 48 h. Ultrasound measurements started after subjects were lying supine on a medical plinth for 10 min. Images were acquired at 55%, 65%, and 75% of the distance between the acromion process of the scapula and the cubital fossa. For image acquisition, the probe was placed perpendicular to the tissue interface, water‐soluble transmission gel was applied over the skin on the region of interest, so measurements were done with caution not to depress the muscle tissue, and images were recorded at individualized field‐of‐view depths. Two experimenters participated in the measurement process so that one handled the probe, and the other was responsible for freezing the images once both considered that image quality was satisfactory. Images were stored in a flash drive and later analyzed using ImageJ software (v. 1.50; NIH, USA). Biceps brachii MT was defined for the three imaged regions (55%, 65%, and 75%; proximal‐distal) as the distance between its superficial and deep aponeuroses. Brachialis was apparent at more distal regions only (65%, 75%), and its MT was defined as the distance between its superficial aponeurosis and the humerus. Inter‐day test‐rest reliability scores were satisfactory (ICC [3,1] = 0.99; relative typical error = 1.2%). Examples of ultrasound images of each region can be seen in the Figure S1.

### 2.4 1RM Strength

Maximum strength was assessed via 1RM test on the respective trained exercise for each arm, as previously done (Maeo et al. 2021, 2023; Stasinaki et al. 2018). For the pre‐training testing session, subjects were randomly selected to start testing for PREA or BAYE, and this was kept the same post‐training individually. Subjects performed a warm‐up of 15 repetitions with approximately 50% of their estimated load to the first attempt, followed by five 1RM attempts. If an attempt was successful or unsuccessful, weight was added or reduced, respectively, for the next attempts by 3%–10% to the nearest 0.5 kg. Subjects were afforded 3–5 min of rest between attempts and 5 min between exercises. Tests were performed with subjects holding the cable handle with their hands supinated, and the shoulder was positioned at about 50° in flexion or extension for PREA and BAYE, respectively. Testing started with the elbow almost fully extended (10°), and subjects were instructed to fully flex it (140°) to complete a valid 1RM attempt. Exercises were performed with the same resistance profiles (Figure 1); the PREA bench and BAYE chair, as well as the pulley height of the training machine, were adjusted individually to ensure similarity between exercises for the arm‐cable starting, right, and ending angles during execution, and the starting hand‐to‐pulley cable distance.

### 2.5 RT Program

Training was performed twice weekly on nonconsecutive days (≥ 48 h apart) in the afternoon hours for 10 weeks, with progression in the number of sets over the weeks (weeks 1–3: 3 sets; 4–7: 4 sets, weeks 8–10: 5 sets). Training intensities of load were initially set relative to 70% 1RM and were adjusted over the sessions so that the subjects could perform the sets in 8–12 RM to (or very close to) failure. The rest between sets was set to 120 s. The PREA and BAYE biceps curls were performed unilaterally, at a tempo of 1:1 s (concentric and eccentric phases, respectively), with shoulder and arm positions and ranges of motion the same as defined for the 1RM tests (Figure 1). The exercise to be trained first was alternated between sessions. Loads were adjusted set by set when concentric failure occurred outside the repetition maximum zone and progressed individually over the weeks to the nearest 0.5 kg. The number of repetitions and the weight used in the first set of every session were recorded to adjust the training load and represent training volume.

### 2.6 Statistical Analyses

Data normality was assessed using the Shapiro–Wilk test, and homogeneity of variances was evaluated with Levene's test. Analyses of variance were used to compare the groups for training measures. For both MT and 1RM, analyses of covariance were performed, with the pre‐to‐post training difference as dependent variables, baseline values as covariates, and group as fixed factors, with adjusted marginal pre‐post mean differences tested against zero to determine training effects relative to baseline. For MT changes, analyses of covariance were performed to compare the groups, whereas analyses of variance were performed to compare percentage changes in MT between muscle regions to examine whether each exercise elicited regional hypertrophy. Bonferroni correction was applied to multiple‐comparison tests. For 1RM changes, given it was hypothesized that improvements would be equivalent between conditions, two one‐sided tests (TOST) for equivalence were performed to compare the groups, standardizing with Hedges' g effect size (ES). The standardized equivalence bounds were set at ± 0.20. ESs were calculated as post‐training mean minus pre‐training mean, divided by pooled pre‐training standard deviation, with Hedges' g correction factor for small samples (Borenstein et al. 2009). An ES of 0.00–0.19 was considered as trivial, 0.20–0.49 as small, 0.50–0.79 as moderate, and ≥ 0.80 as large (Borenstein et al. 2009). A *p* < 0.05 was accepted as statistically significant. The data were expressed as mean, standard deviation, 95% confidence intervals (CIs), average percentage changes, and ESs. Analyses were conducted using JASP v.0.16.2 (JASP Team, Netherlands).

## 3 Results

**FIGURE 2:**
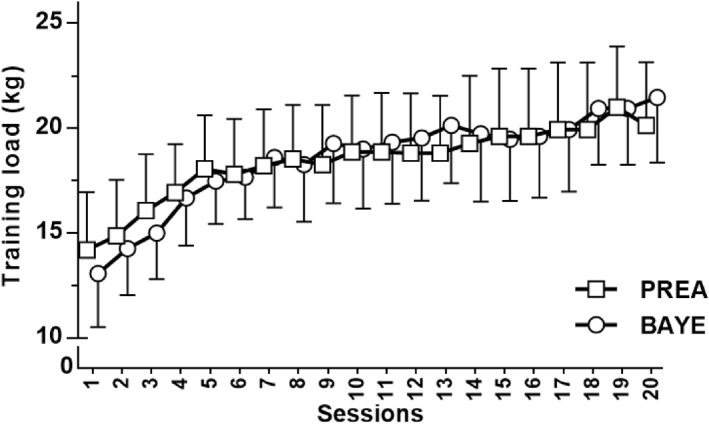
Progression in training load during Preacher (PREA) and Bayesian (BAYE) curl conditions. The horizontal lines are mean and 95% CIs. No difference was detected between groups (comparison of slopes; *p* = 0.252).

Subjects completed all the training sessions. No injuries occurred during the intervention. Training stimuli were matched between conditions, as reflected by the similar average repetitions per set (PREA: 9.5 ± 0.5 repetitions; BAYE: 9.6 ± 0.5 repetitions; *p* = 0.392), the average load (PREA: 18.2 ± 5.0 kg; BAYE: 18.3 ± 4.5 kg; *p* = 0.970), and the weekly progression in load (PREA: 0.5 ± 0.3 kg/wk; BAYE: 0.7 ± 0.4 kg/wk; *p* = 0.252). For 1RM strength, significant (*p* < 0.05) large increases were observed for both conditions (PREA: pre = 21.9 ± 6.7 kg, post = 27.1 ± 6.6 kg; BAYE: pre = 20.7 ± 5.0 kg, post = 28.1 ± 5.9 kg), with equivalent improvements observed between them (PREA: 28% [ES: 0.85], BAYE: 37% [ES: 1.22]; ES difference: 0.37 [95%CIs = −0.33, 1.08]; TOST for equivalence, *p*‐values = 0.061, 0.637). Figure 2 presents the progression in training load by the exercise condition.

**FIGURE 3:**
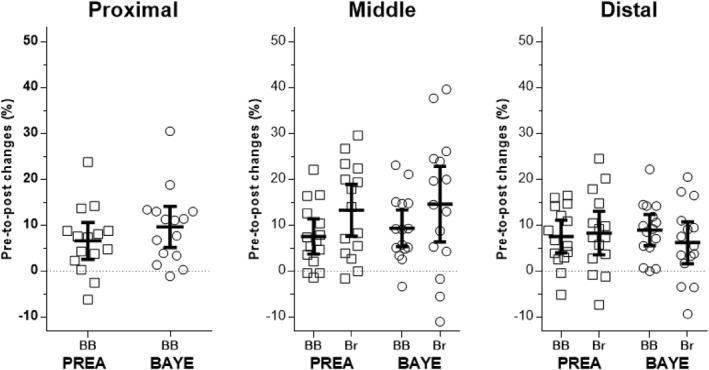
Individual percentage changes from pre‐ to post‐training in muscle thickness (MT) of biceps brachii (BB) and brachialis (Br) for more proximal (55%), middle (65%), and more distal (75%) regions after Preacher (PREA) and Bayesian (BAYE) biceps curls. The horizontal lines are mean and 95% CIs. All measures increased pre‐ to post‐training, but no significant difference was detected between regions or conditions (*p* values = 0.205–0.662).

For muscle thickness (MT), significant (*p* < 0.05) moderate‐to‐large increases were observed at all sites analyzed for both conditions. No significant difference was observed between PREA and BAYE in any muscle regions (*p* values = 0.205–0.662), nor between regions of the biceps brachii for either PREA (*p* = 0.908) or BAYE (*p* = 0.964). Similar responses were observed between conditions for the overall response (sum of the three regions) of the biceps brachii (PREA: 7% [ES: 0.53], BAYE: 9% [ES: 0.68]; *p* = 0.314), and brachialis (PREA: 10% [ES: 0.72], BAYE: 8% [ES: 0.65]; *p* = 0.911), as well as the proximal region (only biceps brachii; PREA: 6% [ES: 0.51], BAYE: 9% [ES: 0.73]; *p* = 0.205), and overall middle (PREA: 8% [ES: 0.65], BAYE: 10% [ES: 0.79]; *p* = 0.374) and distal (PREA: 7% [ES: 0.65], BAYE: 8% [ES: 0.67]; *p* = 0.946) regions, or the sum of all measured regions (PREA: 8% [ES: 0.66], BAYE: 9% [ES: 0.77]; *p* = 0.416). While comparing relative changes between muscles, no significant difference was detected between biceps brachii and brachialis for PREA (7% [ES: 0.53] vs. 10% [ES: 0.72]; *p* = 0.325) or BAYE (9% [ES: 0.68] vs. 8% [ES: 0.65]; *p* = 0.895). Table 1 displays the results of pre‐ and post‐training data and Figure 3 presents the individual relative changes, according to conditions, muscles, and regions.

**TABLE 1:** Training effects on regional muscle thickness (mm) after Preacher (PREA) and Bayesian (BAYE) biceps cable curls.

	PREA (*n* = 15)	BAYE (*n* = 15)
Proximal—Biceps brachii		
Pre	28.2 ± 3.2	28.5 ± 3.7
Post	30.0 ± 3.2^*^	31.0 ± 3.3^*^
ES	0.51 (0.14, 0.88)	0.73 (0.34, 1.13)
Middle—Biceps brachii		
Pre	24.5 ± 3.6	24.2 ± 3.3
Post	26.2 ± 3.2^*^	26.4 ± 2.9^*^
ES	0.49 (0.13, 0.86)	0.62 (0.24, 1.00)
Distal—Biceps brachii		
Pre	23.5 ± 3.3	23.0 ± 3.0
Post	25.2 ± 3.0^*^	25.0 ± 2.8^*^
ES	0.53 (0.16, 0.90)	0.62 (0.24, 1.00)
Middle—Brachialis		
Pre	5.7 ± 0.7	6.0 ± 1.0
Post	6.5 ± 0.8^*^	6.8 ± 1.2^*^
ES	0.84 (0.44, 1.24)	0.94 (0.52, 1.36)
Distal—Brachialis		
Pre	11.1 ± 1.5	11.5 ± 1.6
Post	11.9 ± 1.7^*^	12.1 ± 1.6^*^
ES	0.57 (0.19, 0.94)	0.42 (0.06, 0.78)

*Notes:* Data are mean ± standard deviation. Effect sizes (ES) are presented as mean and 95% confidence intervals (upper, lower bounds).

^*^ *p* < 0.05 versus Pre. No significant difference was detected between groups.

## 4 Discussion

In the present study, the effects of RT on regional hypertrophy and maximum strength of the elbow flexor muscles were compared between elbow flexion exercises performed with different shoulder positions (flexion [PREA] vs. extension [BAYE]). It was hypothesized that biceps brachii hypertrophy would be greater for BAYE than PREA, but changes in strength would be equivalent between conditions. After the intervention, similar adaptations were observed following PREA and BAYE biceps curls, rejecting the hypothesis for hypertrophy, but supporting the hypothesis on the strength gains.

Altering shoulder position may have shortened the biceps brachii fibers in PREA and lengthened them in BAYE, potentially shifting the sarcomere operating length along the force–length curve, with BAYE operating more on optimal lengths for force production (Koo et al. 2002; Murray et al. 2000; Newham et al. 1988). However, it seems that such an advantage for BAYE did not occur since the exercises were performed with similar weights throughout the intervention (Figure 2), resulting in similar torques between conditions (assuming: (i) the exercises were matched in torque‐angle profiles, as shown in Figure 1; and (ii) the exercises were performed at similar execution velocities). The *post hoc* elaboration of the force–length relationship model (Figure 1) supports the idea that such a variation in shoulder position likely elicited only small changes in biceps fiber operating lengths during training, and this may explain the lack of differential biceps growth between exercises. Moreover, changes in tendon properties after a few training sessions may impact tendon slack length, an effect that is influenced by the muscle‐length training position (Lambrianides et al. 2024), thereby affecting muscle–tendon behavior during exercise, deviating from the predicted models. Additionally, there was no emphasis on the lengthening phase of the contraction. Performing the eccentric phase at a slower tempo (e.g., 4 s) could have allowed BAYE to experience more time under higher loads in longer lengths, potentially providing an advantage over PREA. It remains to be explored whether manipulating execution tempo influences the effects of longer‐length RT on muscle growth.

Korta et al. (2023) and others (Kassiano et al. 2025; Kobayashi et al. 2024; Vendruscolo et al. 2024) have recently compared exercises similar to the ones used herein, where they used biceps dumbbell curls that have opposite resistance profiles: one emphasizing torque at the end of the eccentric phase but pre‐shortening the biceps by shoulder flexion (a similar version of “PREA” used here, but done with dumbbells), and the other emphasizing torque at the end of the concentric phase but pre‐lengthening the biceps by shoulder extension (“BAYE” done with dumbbells [incline curl]). Therefore, inferences on the effects of longer versus shorter muscle length training could not be made from such comparisons as two factors distinguished their exercises: resistance exercise profiles and shoulder positions. Moreover, hypertrophy measures were often taken for the “elbow flexors”; whether the observed differential results stemmed from biceps or brachialis hypertrophy could not be established. In addition, Nunes et al. (2020) compared exercises emphasizing greater torques at shorter or longer muscle lengths with the same shoulder position (flexed; cable vs. dumbbell PREA) and found that no difference in biceps brachii MT increases; however, the biceps was measured only at the mid‐segment region. In this regard, the influence of training at shorter versus longer biceps brachii lengths on regional changes in biceps size was unclear, and the present study is the first where biceps brachii MT changes were measured regionally while isolating differences in muscle length as a factor to be compared between exercises, revealing no significant difference between conditions.

Interestingly, other studies have reported greater distal arm muscle growth after dumbbell PREA curls but did not measure biceps brachii size isolated from the brachialis (Kassiano et al. 2025; Korta et al. 2023; Sato et al. 2021). Therefore, the increased growth in more distal regions might be attributed to enhanced brachialis development, not to a biceps regional hypertrophy. In the present study, a *post hoc* analysis of the biceps brachii “net hypertrophy”, calculated as the biceps relative change minus brachialis relative changes, revealed a small difference (ES = 0.38) favoring BAYE (biceps–brachialis; PREA: 7%–10%, BAYE: 9%–8%), suggesting a shift in muscle recruitment preference for the biceps in BAYE compared to PREA. Supporting this idea, BAYE resulted in next to no negative changes in MT across all three biceps regions, as can be seen in individual results presented in Figure 3. Moreover, in a recent nonpeer‐reviewed work presented as a conference abstract (Kobayashi et al. 2024), authors reported that dumbbell PREA induced greater increases in brachialis size, whereas dumbbell BAYE induced greater increases in biceps brachii size.

In addition, no study has reported that the biceps brachii exhibit inhomogeneous regional hypertrophy, despite presenting regional strains during shortening (Blemker et al. 2005; Pappas et al. 2002). Different strains across regions of the fascicles could cause different regions of the muscle to experience varying mechanical work and, thus, receive different stimuli for growth (Jorgenson et al. 2020). However, different strains alone may not explain potential inhomogeneous regional hypertrophy. It is known that skeletal muscles exhibit regional variations in resting sarcomere lengths across muscle regions and even within the same fiber, and sarcomeres in different muscle regions have their own operating lengths (Jorgenson et al. 2020; Pappas et al. 2002; Purslow 2010). Of note, sarcomeres experiencing greater strain but operating in regions of lower tension on the force–length curve might experience reduced stimuli for growth. Due to the pre‐shortening state in PREA, the more proximal regions of the biceps were expected to be insufficiently active and then receive less tension stimulus for growth than the distal regions, and then BAYE for the same region. However, these were not observed herein. It is possible that the biceps brachii architecture permits homogeneous force production and transmission across its fiber bundles, and altering shoulder position does not affect how stimuli are set to it during maximum elbow flexion exercises. It is important to note that the present study lasted 10 weeks, and a potential difference might emerge over a longer timeframe, warranting further investigation.

Similar results between conditions were also observed for 1RM strength gains. These findings can be attributed to the comparable loads used and their relatively similar progression across sessions. Since 1RM testing was conducted only for the specific exercise each arm performed, the influence of training‐test specificity limits inferences about the muscle's overall force production capacity (Buckner et al. 2017). Nonetheless, this testing design followed previous works, which also showed similar relative increases in strength between different exercises trained at shorter versus longer lengths (Maeo et al. 2021, 2023; Stasinaki et al. 2018). The effects on nonspecific strength tests remain unknown. Results from Sato et al. (2021) suggest greater “global” strength increases (as evaluated by varied testing modes) with longer‐length training. Further investigations are warranted exploring these aspects as well as the neuroplasticity of the strength adaptation (Škarabot et al. 2021). Of additional note, any potential cross‐education interference effect on maximum strength (i.e., improvements in the contralateral, traditionally nontrained, and arm) appears negligible in this context, as it is unlikely to occur when both limbs are trained, particularly with similar loads (Song et al. 2024).

The present study has some limitations. Although muscle thickness was assessed in two distinct regions of the limb, as recommended (Nunes et al. 2024), measuring cross‐sectional area at sites closer to the arm extremities (Drummond et al. 2016) or evaluating each biceps brachii head separately could have provided additional insights, particularly given their distinct force–length relationships (Koo et al. 2002). Additionally, although participants were carefully instructed and supervised to maintain the specified shoulder positions (visually verified after extensive piloting using mobile device‐based goniometers) and to perform exercises with consistent ranges of motion and tempos, continuous monitoring with specialized devices (e.g., electrogoniometers and metronomes) was not implemented.

## 5 Conclusion

In conclusion, biceps cable curl training with the shoulder flexed (PREA) or extended (BAYE) resulted in similar increases in muscle size and strength. Altering shoulder position does not appear to influence the effects of biceps cable curls after 10 weeks of RT in untrained young men. There is no evidence that the biceps brachii benefit from longer‐length RT or exhibit regional hypertrophy.

## Author Contributions

P.A. was involved with conceptualization, methodology, and data collection. J.P.N. analyzed the data, generated the figures, and drafted the first version of the manuscript. S.K., S.N., A.G., H.N., S.N., S.M., and R.S. participated in the data collection and provided critical revisions to the writing. K.N. provided critical revisions to the manuscript. All authors read, revised critically, and approved the final version of the manuscript.

## Ethics Statement

The present study was conducted according to the Declaration of Helsinki. The present study was approved by the University of Tehran Ethics Review Board Committee.

## Conflicts of Interest

The authors declare no conflicts of interest.

## Supporting information

Figure S1
