# Supplementary material for: Comparison Between Shoulder Flexed and Extended Positions in Elbow Flexion Resistance Training on Regional Hypertrophy and Maximum Strength: Preacher versus Bayesian Cable Curls
Source: Eur J Sport Sci. 2025 Mar 13;25(4):e12279. doi: 10.1002/ejsc.12279 (PMC11906226; doi:10.1002/ejsc.12279)
Supplement: Supplementary file 1 — Figure S1 [file EJSC-25-e12279-s001.pdf]

Supplementary Material

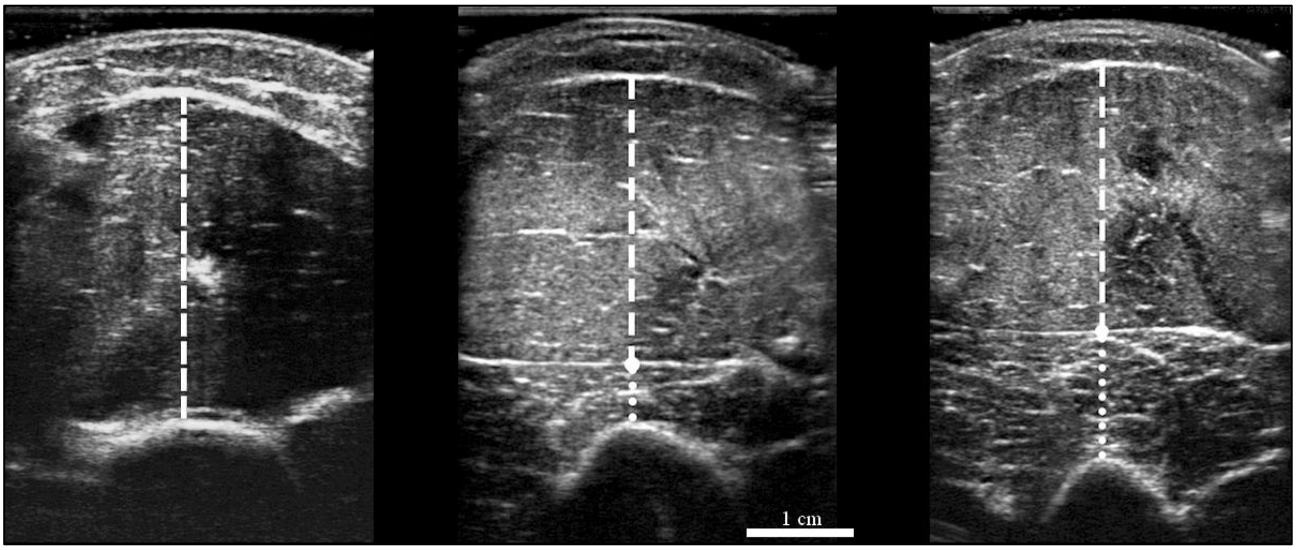

S1. Overview of the measure of the thickness of elbow flexors. Left to right: proximal, middle, and distal measures. Long dash line: Biceps brachii. Dotted line: Brachialis.
